# White blood cells, monocytes and thrombin time in predicting symptomatic hydrocephalus in patients with aneurysmal subarachnoid hemorrhage

**DOI:** 10.3389/fsurg.2025.1598385

**Published:** 2025-07-02

**Authors:** Hui Deng, Hongjuan Yang, Ruoyu Chen, Wei Xing, Jia Shi

**Affiliations:** ^1^ Department of Neurosurgery, The Third Affiliated Hospital of Soochow University, Changzhou, China; ^2^ Department of Endocrinology, The Third Affiliated Hospital of Soochow University, Changzhou, China; ^3^ Department of Imaging, The Third Affiliated Hospital of Soochow University, Changzhou, China

**Keywords:** aneurysmal subarachnoid hemorrhage, acute symptomatic hydrocephalus, white blood cells, monocytes, thrombin time

## Abstract

**Objective:**

To investigate the value of admission blood routine and coagulation function parameters in predicting acute symptomatic hydrocephalus in patients with aneurysmal subarachnoid hemorrhage (aSAH).

**Methods:**

This retrospective study included 423 patients with aSAH admitted to the Department of Neurosurgery of the Third Affiliated Hospital of Soochow University from November 2013 to September 2020. Demographic, clinical and laboratory data were collected. The patients were divided into hydrocephalus group (*n* = 96) and non-hydrocephalus group (*n* = 327) according to the presence of hydrocephalus on the first head CT. Univariate and multivariate logistic regression analyses were used to determine the independent risk factors for acute symptomatic hydrocephalus after aSAH.

**Results:**

Among the 423 aSAH patients, 96 (22.70%) developed acute symptomatic hydrocephalus. Multivariate logistic regression analysis showed that, after adjusting for confounding factors, white blood cells (WBC) (OR = 1.121, 95% CI = 1.067–1.181), monocytes (M) (OR = 2.812, 95% CI = 1.183–6.699), and thrombin time (TT) (OR = 0.843, 95% CI = 0.729–0.948) were independently associated with the development of hydrocephalus. Further analysis of the area under the receiver operating characteristic (ROC) curve indicated that, compared to basic clinical data, the combined prediction model of “basic clinical data + WBC + M + TT” performed better (AUC = 0.728, 95% CI = 0.682–0.769, *P* = 0.004).

**Conclusions:**

The WBC, M and TT within 24 h of admission in aSAH patients can be used to predict the occurrence of acute symptomatic hydrocephalus.

## Introduction

Spontaneous subarachnoid hemorrhage (SAH) is one of the most common types of stroke and is often associated with aneurysm rupture. An intracranial aneurysm refers to a localized, pathological dilation of the intracranial arterial wall, which carries a risk of rupture and is the leading cause of aneurysmal subarachnoid hemorrhage (aSAH) (1). According to statistics, the incidence of aSAH accounts for approximately 5% of all stroke cases worldwide, with a mortality rate of 35% (1, 2). Hydrocephalus is a common complication in patients with aSAH and a risk factor for neurological dysfunction, cognitive impairment, and urinary incontinence, seen in approximately 12%–31% of patients with aSAH (3). Hydrocephalus in SAH can be classified into acute (0–3 days post-SAH), subacute (4–13 days post-SAH), and chronic (more than 14 days post-SAH) stages (4), with cerebrospinal fluid circulation disorders being the primary cause. Early identification of risk factors for the development of hydrocephalus is crucial for guiding clinical treatment, improving patient prognosis, and reducing mortality (5).

Immune-mediated inflammatory responses were critical determinants of early brain injury following aSAH (6). Increasing evidence suggested that white blood cells after aSAH associated positively with the severity of the disease (7–9). Monocyte counts tended to rise during the early stages of brain injury (10), and these cells played an active protective role by clearing debris from the subarachnoid space (11). Furthermore, levels of thrombin and fibrinolytic components were elevated in the cerebrospinal fluid (CSF) of SAH patients (12). Obstruction of CSF circulation, caused by blood clots and tissue factors from brain injury, were associated with pathological conditions such as hydrocephalus (13). While the roles of these hematological markers in the course of aSAH had been extensively reported, their specific biochemical interactions had not been thoroughly explored in the existing literature. Moreover, current research tends to focus on single laboratory markers, lacking a comprehensive multidimensional and multifactorial assessment.

In this study, we retrospectively analyzed the clinical data of 423 patients with aSAH at admission, studied the correlation between the first blood routine and coagulation function parameters and acute symptomatic hydrocephalus in patients with aSAH within 24 h of admission, and further explored the predictive value of blood routine and coagulation function parameters for acute symptomatic hydrocephalus in patients with aSAH. The objective was to provide guidance for early clinical intervention, diagnosis, and treatment of secondary acute symptomatic hydrocephalus in aSAH patients.

## Methods

### Study selection

**Figure 1:**
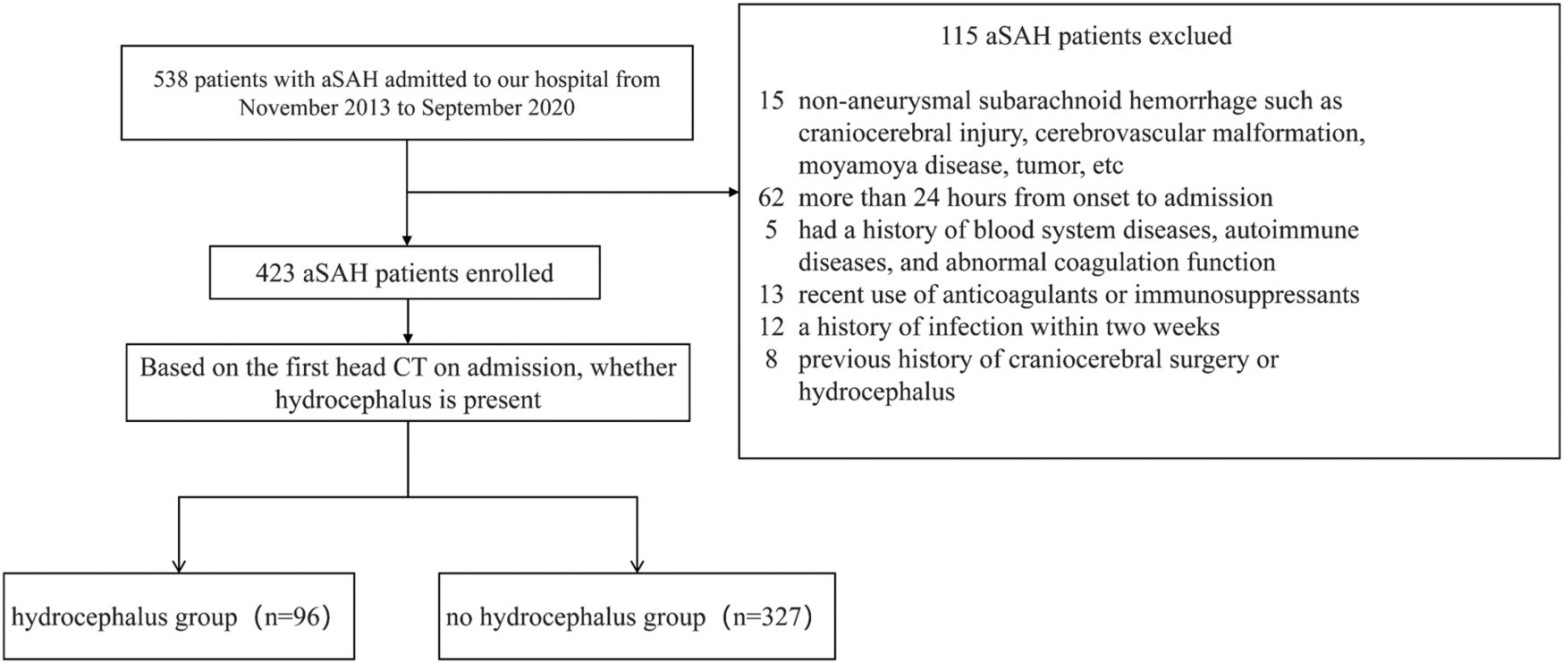
Patient selection flow chart. aSAH, aneurysmal subarachnoid hemorrhage.

A total of 423 patients with aSAH were admitted to the Department of Neurosurgery of the Third Affiliated Hospital of Soochow University from November 2013 to September 2020 were selected as the study participants (Figure 1). Inclusion criteria: (1) SAH was confirmed by CT at admission and there was no history of trauma; (2) Digital subtraction angiography (DSA), CT angiography (CTA) or surgical diagnosis of subarachnoid hemorrhage caused by cerebral aneurysm rupture; (3) Blood sample taken upon admission or within 24 h for laboratory testing; (4) Aneurysm interventional embolization was performed within 48 h after admission; (5) Patients who were at least 18 years of age with complete clinical data. Exclusion criteria: (1) patients with brain injury, cerebrovascular malformation, moyamoya disease, tumor and other non-aneurysmal subarachnoid hemorrhage; (2) Patients with more than 24 h from onset to admission; (3) Patients with a history of blood system diseases, autoimmune diseases, and abnormal coagulation function; (4) Patients who have recently used anticoagulants or immunosuppressants; (5) Patients with a history of infection within two weeks; (6) Patients with a history of hydrocephalus.

This study was approved by the Medical Ethics Committee of the Third Affiliated Hospital of Soochow University. Given the retrospective nature of the study, we waived informed consent.

### Diagnosis of acute symptomatic hydrocephalus

Acute symptomatic hydrocephalus following aSAH includes both clinical manifestations (such as altered mental status, drowsiness, or pupil changes) and CT findings (such as ventricular enlargement, increased temporal horn size, disappearance of the cisterns, and effacement of the sulci) (14). This condition not only exacerbates early neurological deficits in aSAH patients, further aggravating clinical symptoms, but also negatively impacts neurological recovery during the postoperative period (15). Management in both the acute and subacute phases typically requires the placement of external ventricular drainage (EVD) to mitigate the harmful secondary effects following aneurysmal rupture (16).

### Data collection

General data of study patients were collected from the electronic medical record system, including age, sex, alcohol and smoking history, whether they had underlying medical conditions (hypertension, coronary heart disease, diabetes, cerebral infarction), and Hunt-Hess rating was assessed upon admission; Blood routine and coagulation function indexes were collected within 24 h after admission. Minimal missing values (<2% for lab parameters) were handled via complete-case analysis. Sensitivity analyses confirmed result consistency. Blood routine tests were performed using a Sysmex XN-9000 analyzer (Sysmex Corporation), and coagulation parameters were measured on an STA-R MAX system (Stago). All assays adhered to standardized laboratory reference ranges, with strict quality control (intra-assay CV <4%, inter-assay CV <6%). The patients were divided into hydrocephalus group (*n* = 96) and non-hydrocephalus group (*n* = 327) for comparison according to head CT and symptomology. All investigators were uniformly trained and passed assessments.

### Statistical analysis

In this study, R 4.3.1 and SPSS 25 were used for statistical analysis. Categorical variables were expressed as frequency (percentage) and continuous variables were expressed as mean ± standard deviation (SD) or median (interquartile distance) depending on the distribution. The total population was divided into two groups according to whether hydrocephalus was present or not: the hydrocephalus-free group and the hydrocephalus group. Baseline characteristics between the two groups were compared. An independent samples t-test or rank-sum test was used for continuous variables, and the chi-square test was used for categorical variables. Logistic regression was used to analyze the correlation between blood routine and coagulation function parameters and hydrocephalus group. Model 1 was adjusted for age and sex. Previous studies have shown that lifestyle is an important influencing factor in hydrocephalus group, especially smoking and drinking (17), so model 2 further adjusted smoking and drinking. The history of some common underlying diseases was also an important influencing factor for the hydrocephalus group (8). Based on model 2, Model 3 further adjusted hypertension, diabetes, coronary heart disease and cerebral infarction. Receiver Operating Characteristic (ROC) curves were plotted to evaluate the ability of blood routines and coagulation function parameters to diagnose hydrocephalus. The Net Reclassification Improvement (NRI) and Integrated Discrimination Improvement (IDI) were also calculated to estimate the incremental predictive value of combined hemocyte and coagulation function parameters compared to the underlying clinical data. In addition, the analysis was stratified according to sex and age to eliminate the interference of confounding factors. The cut-off point for age stratification was 60 years, dividing patients into <60 years old and ≥60 years old.

## Results

### Basic characteristics of study subjects

In this study, a total of 538 patients with aSAH were enrolled, of whom 115 were excluded, leaving 423 subjects included in the final analysis. The median age was 57 years, with 164 males, accounting for 37.96%, and 96 patients with acute symptomatic hydrocephalus, accounting for 22.70%. According to whether hydrocephalus was present (Table 1), the results showed that compared with the group without hydrocephalus, patients in the hydrocephalus group were older (*P* = 0.002) and had higher concentrations of white blood cells (WBC), neutrophils, monocytes (M), neutrophil-to-lymphocyte ratio (NLR) and fibrinogen (FIB) (all *P* < 0.05). Thrombin time (TT) was lower (*P* = 0.002). In addition, patients in the hydrocephalus group were more likely to have hypertension (HTN), cerebral infarction (CI), intraventricular hemorrhage (IVH), and higher Hunt-Hess scores (*P* < 0.05).

**Table 1:** Baseline characteristics of participants.

Variables	Total	Non-hydrocephalus	Hydrocephalus	*P*-value
*N*	423	327	96	
Gender				
Male	164 (37.96%)	125 (38.23%)	39 (40.63%)	0.671
Female	259 (61.23%)	202 (61.77%)	57 (59.38%)	
Age	57 (50–67)	56 (50–65)	63 (53–69.75)	0.002
Smoking	30 (7.09%)	21 (3.06%)	9 (9.38%)	0.322
Drinking	18 (4.26%)	16 (4.89%)	2 (2.08%)	0.230
WBC	11.82 (9.18–15.28)	11.4 (8.8–14.28)	13.49 (10.33–18.57)	<0.001
Neutrophil	9.65 (6.73–12.79)	9.13 (6.4–12.17)	11.85 (8.09–16.52)	<0.001
Lymphocyte	1.21 (0.82–2.1)	1.2 (0.85–1.93)	1.21 (0.78–2.28)	0.820
M	0.46 (0.32–0.67)	0.45 (0.31–0.62)	0.55 (0.34–0.77)	0.025
PLT	206 (168–244)	205 (169–243)	209 (164.25–255.0)	0.493
MPV	10.7 (10.0–11.6)	10.7 (11.3–14.5)	10.8 (10.1–11.78)	0.151
PDW	12.6 (11.3–14.7)	12.6 (11.3–14.5)	12.8 (11.5–15.28)	0.144
NLR	8.13 (3.96–13.97)	7.98 (3.77–12.73)	9.84 (6.05–15.09)	0.026
NMR	19.95 (13.62–30.1)	19.8 (13.40–29.72)	20.39 (15.40–35.29)	0.154
MLR	0.35 (0.22–0.54)	0.34 (0.22–0.52)	0.39 (0.20–0.57)	0.332
MPV/PLT	0.05 (0.04–0.07)	0.05 (0.04–0.07)	0.05 (0.04–0.07)	0.818
PDW/PLT	0.06 (0.05–0.08)	0.06 (0.05–0.08)	0.06 (0.05–0.09)	0.786
PT	11.2 (10.6–11.8)	11.3 (10.6–11.8)	11.2 (10.6–11.9)	0.844
INR	0.97 (0.91–1.02)	0.97 (0.91–1.02)	0.97 (0.9–1.02)	0.732
PTR	0.97 (0.91–1.02)	0.97 (0.91–1.02)	0.97 (0.91–1.02)	0.713
APTT	25.4 (23.9–27.3)	25.4 (23.9–27.3)	25.3 (23.73–26.98)	0.356
FIB	2.51 (2.1–3.0)	2.47 (2.06–2.90)	2.68 (2.32–3.22)	0.012
TT	18.4 (17.0–19.3)	18.5 (17.2–19.5)	18.0 (16.33–18.70)	0.002
HTN	288 (68.09%)	211 (64.53%)	77 (80.21%)	0.004
CHD	16 (3.78%)	11 (3.36%)	5 (5.21%)	0.405
DM	31 (7.33%)	20 (6.12%)	11 (11.46%)	0.077
CI	32 (7.66%)	19 (5.81%)	13 (13.54%)	0.012
IVH	207 (48.94%)	117 (35.78%)	90 (93.75%)	<0.001
Hunt-Hess grade				
<3	201 (47.52%)	189 (57.8%)	12 (12.5%)	<0.001
≥3	222 (52.48%)	138 (42.2%)	84 (87.5%)	

WBC, white blood cell; M, monocytes; PLT, platelet; MPV, mean platelet volume; PDW, platelet distribution width; NLR, neutrophil to lymphocyte ratio; NMR, neutrophil to monocytes ration; MLR, monocytes to lymphocyte ratio; MPV/PLT, MPT to PLT ratio; PDW/PLT, PDW to PLT ratio; PT, prothrombin time; INR, international normalized ratio; PTR, partial thromboplastin time ratio; APTT, activated partial thromboplastin time; FIB, fibrinogen; TT, thrombin time; HTN, hypertension; CHD, coronary atherosclerotic heart disease; DM, diabetes mellitus; CI, cerebral infarction; IVH, intraventricular hemorrhage.

### Correlation of blood routine and coagulation parameters with hydrocephalus group

When confounding factors were not adjusted, WBC, neutrophils and M, and coagulation function parameters FIB and TT were statistically significant elevated in the hydrocephalus group compared with the non-hydrocephalus group. After further adjusting for potential confounders (Model 3), WBC (OR = 1.121, 95% CI = 1.067–1.181), M (OR = 2.812, 95% CI = 1.183–6.699) and TT (OR = 0.843, 95% CI = 0.729–0.948) were independently associated with the formation of hydrocephalus. The results of neutrophils were not statistically significant (Table 2). WBC, M, and TT were categorized to further analyze their correlation with the hydrocephalus group (Table 3). The results showed that patients in the highest quartile of WBC had a significantly increased risk of aSAH complicated with acute symptomatic hydrocephalus compared with those in the lowest quartile. The risk of M in the upper quartile also tended to increase, but there was no statistical significance in model 3. Conversely, the risk of aSAH complicated with acute symptomatic hydrocephalus was significantly decreased for TT in the highest quartile. It is worth noting that when stratified analysis was performed, we found significant associations between WBC, M, and TT and acute symptomatic hydrocephalus with aSAH in different age and sex subgroups (Table 4). WBC and M may lead to a greater risk of aSAH with acute symptomatic hydrocephalus in men and patients <60 years of age. TT may lead to a lower risk of aSAH complicated with acute symptomatic hydrocephalus in women and patients ≥60 years of age.

**Table 2:** Logistic regression analysis of blood routine and coagulation function and aSAH with acute symptomatic hydrocephalus.

Variants	Univariate logistic regression		Multivariate logistic regression	
	OR (95% CI)	*P*-value	OR (95% CI)	*P*-value
WBC	1.123 (1.07–1.181)	<0.001	1.121 (1.067–1.181)	<0.001
Neutrophil	1.029 (1.005–1.063)	0.035	1.027 (1.003–1.061)	0.053
M	3.258 (1.429–7.444)	0.005	2.812 (1.183–6.699)	0.019
Lymphocyte	1.003 (0.85–1.165)	0.971		
NLR	1.015 (0.998–1.034)	0.094		
NMR	1.003 (0.997–1.008)	0.322		
MLR	1.82 (0.977–3.353)	0.054		
PLT	1.002 (0.998–1.005)	0.350		
PDW	1.001 (0.959–1.026)	0.967		
MPV	0.992 (0.885–1.042)	0.807		
MPV/PLT	0.799 (0.001–81.712)	0.932		
PDW/PLT	1.157 (0.033–15.844)	0.920		
PT	0.936 (0.737–1.158)	0.566		
INR	0.462 (0.03–5.561)	0.562		
PTR	0.457 (0.027–5.886)	0.568		
FIB	1.389 (1.028–1.881)	0.032	1.318 (1.047–1.792)	0.062
TT	0.849 (0.747–0.959)	0.010	0.843 (0.729–0.948)	0.007
APTT	0.958 (0.884–1.021)	0.259		

Factors corrected for in multifactor logistic regression include: age, gender, smoking, drinking, hypertension, coronary atherosclerotic heart disease, diabetes mellitus, cerebral infarction.

OR, odds ratio; CI, confidence interval; WBC, white blood cell; M, monocytes; PLT, platelet; MPV, mean platelet volume; PDW, platelet distribution width; NLR, neutrophil to lymphocyte ratio; NMR, neutrophil to monocytes ration; MLR, monocytes to lymphocyte ratio; MPV/PLT, MPT to PLT ratio; PDW/PLT, PDW to PLT ratio; PT, prothrombin time; INR, international normalized ratio; PTR, partial thromboplastin time ratio; APTT, activated partial thromboplastin time; FIB, fibrinogen; TT, thrombin time.

**Table 3:** Multivariate logistic regression analysis of categorical variants and aSAH with acute symptomatic hydrocephalus.

Variants	Model 1		Model 2		Model 3	
	OR (95% CI)	*P*-value	OR (95% CI)	*P*-value	OR (95% CI)	*P*-value
WBC						
Quartiles						
Q1 (<9.18)	Ref.	–	Ref.	–	Ref.	–
Q2 (9.18–11.82)	1.531 (0.713–3.288)	0.275	1.425 (0.658–3.089)	0.369	1.313 (0.600–2.875)	0.496
Q3 (11.82–15.28)	1.741 (0.819–3.699)	0.149	1.652 (0.774–3.528)	0.194	1.159 (0.702–3.284)	0.288
Q4 (>15.28)	4.555 (2.246–9.238)	<0.001	4.358 (2.142–8.866)	<0.001	4.116 (1.994–8.499)	<0.001
M						
Quartiles						
Q1 (<0.32)	Ref.	–	Ref.	–	Ref.	–
Q2 (0.32–0.46)	0.968 (0.483–1.940)	0.926	0.942 (0.467–1.899)	0.868	0.983 (0.484–1.997)	0.962
Q3 (0.46–0.67)	1.041 (0.525–2.064)	0.908	0.979 (0.489–1.961)	0.953	0.940 (0.466–1.895)	0.862
Q4 (>0.67)	2.040 (1.060–3.925)	0.033	2.000 (1.034–3.867)	0.039	1.938 (0.991–3.789)	0.053
TT						
Quartiles						
Q1 (<17)	Ref.	–	Ref.	–	Ref.	–
Q2 (17–18.4)	0.839 (0.460–1.532)	0.568	0.837 (0.455–1.537)	0.566	0.800 (0.431–1.486)	0.481
Q3 (18.4–19.3)	0.585 (0.308–1.112)	0.102	0.544 (0.283–1.043)	0.067	0.504 (0.259–0.978)	0.043
Q4 (>19.3)	0.382 (0.188–0.775)	0.008	0.376 (0.183–0.773)	0.008	0.336 (0.160–0.707)	0.004

OR, odds ratio; CI, confidence interval; WBC, white blood cell; M, monocytes; TT, thrombin time; Ref, reference.

Model 1: Adjusted age, gender.

Model 2: Adjusted age, gender, smoking, drinking.

Model 3: Adjusted age, gender, smoking, drinking, Hypertension, Coronary atherosclerotic heart disease, Diabetes mellitus, Cerebral infarction.

**Table 4:** Stratified analysis of blood routine and coagulation function and aSAH with acute symptomatic hydrocephalus.

Variants	Model 1		Model 2		Model 3	
	OR (95% CI)	*P*-value	OR (95% CI)	*P*-value	OR (95% CI)	*P*-value
WBC						
Gender						
Male	1.173 (1.076–1.289)	<0.001	1.173 (1.074–1.291)	<0.001	1.181 (1.076–1.308)	<0.001
Female	1.106 (1.044–1.175)	<0.001	1.104 (1.043–1.174)	<0.001	1.098 (1.037–1.167)	0.002
Age						
Age <60	1.135 (1.061–1.223)	<0.001	1.132 (1.057–1.221)	<0.001	1.128 (1.054–1.216)	<0.001
Age ≥60	1.110 (1.035–1.193)	0.004	1.108 (1.033–1.191)	0.005	1.101 (1.024–1.188)	0.011
M						
Gender						
Male	5.900 (1.641–22.666)	0.007	6.080 (1.612–24.843)	0.009	5.761 (1.501–24.098)	0.012
Female	1.940 (0.630–5.970)	0.248	1.889 (0.613–5.827)	0.268	1.570 (0.491–5.018)	0.446
Age						
Age <60	9.780 (2.880–35.618)	<0.001	10.205 (2.891–39.196)	<0.001	9.559 (2.665–37.350)	<0.001
Age ≥60	1.064 (0.317–3.430)	0.918	0.994 (0.291–3.243)	0.993	0.812 (0.228–2.731)	0.740
TT						
Gender						
Male	0.884 (0.719–1.082)	0.232	0.864 (0.697–1.066)	0.175	0.854 (0.681–1.064)	0.162
Female	0.845 (0.716–0.984)	0.039	0.847 (0.722–0.994)	0.042	0.828 (0.702–0.978)	0.026
Age						
Age <60	0.874 (0.719–1.063)	0.176	0.850 (0.695–1.039)	0.113	0.839 (0.862–1.029)	0.092
Age ≥60	0.860 (0.724–1.003)	0.071	0.862 (0.723–1.008)	0.081	0.819 (0.677–0.970	0.031

OR, odds ratio; CI, confidence interval; WBC, white blood cell; M, monocytes; TT, thrombin time; Ref, reference.

Model 1: Adjusted age, gender.

Model 2: Adjusted age, gender, smoking, drinking.

Model 3: Adjusted age, gender, smoking, drinking, Hypertension, Coronary atherosclerotic heart disease, Diabetes mellitus, Cerebral infarction.

### Evaluation of diagnostic efficacy of blood routine and coagulation parameters in patients with aSAH complicated with acute symptomatic hydrocephalus

**Figure 2:**
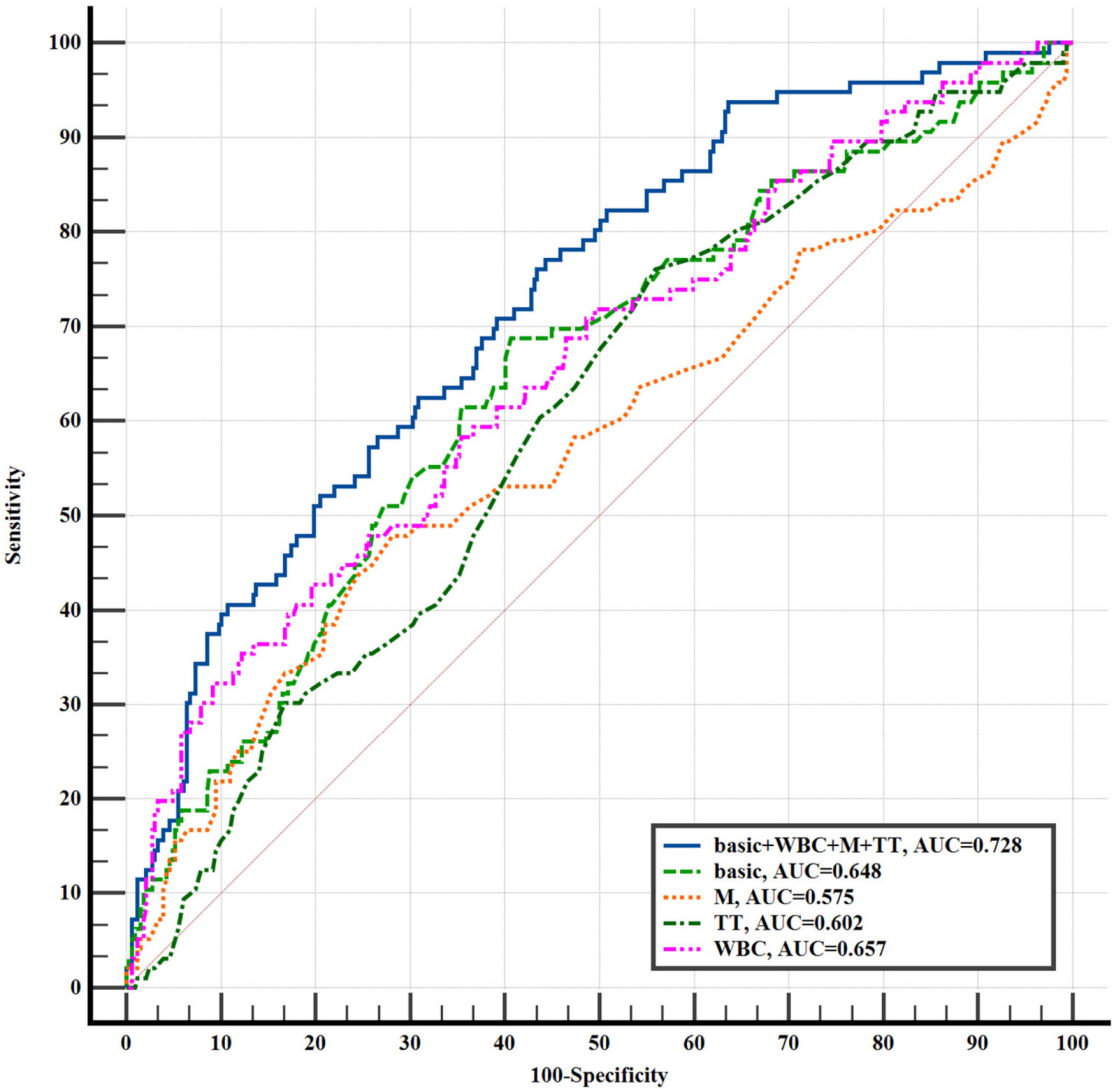
The ROC curve of aSAH with acute symptomatic hydrocephalus was predicted by each indicator. basic: age, gender, drinking, smoking, hypertension, coronary atherosclerotic heart disease, diabetes mellitus, cerebral infarction; WBC, white blood cell; M, monocytes; TT, thrombin time.

We plotted the ROC curve to evaluate the predictive performance of WBC, M, and TT for the development of acute symptomatic hydrocephalus in aSAH patients (Figure 2). As shown in Figure 2, the combined predictive model yielded an AUC of 0.728 (95% CI = 0.682–0.769, *P* = 0.004), indicating moderate discriminative ability and a statistically significant improvement over basic clinical data alone. The optimal clinical thresholds derived from ROC analysis (maximizing Youden's index) were: WBC: 9.18 × 10^9^/L (sensitivity 35.4%, specificity 87.8%); Monocytes: 0.46 × 10^9^/L (sensitivity 47.9%, specificity 71.9%); TT: 18.4 s (sensitivity 76.0%, specificity 44.0%). At these thresholds, this model achieved a balanced sensitivity and specificity, supporting its clinical applicability for early risk identification. These findings illustrate that incorporating WBC, M, and TT into the model enhances the accuracy of predicting acute symptomatic hydrocephalus in aSAH patients. Furthermore, adding WBC, M, and TT to the basic clinical data significantly improved the diagnostic performance for acute symptomatic hydrocephalus in aSAH patients compared to the basic clinical data alone, as evidenced by a continuous NRI of 0.490 (95% CI = 0.268–0.712, *P* < 0.001), categorical NRI of 0.170 (95% CI = 0.058–0.282, *P* = 0.003), and IDI of 0.070 (95% CI = 0.041–0.098, *P* < 0.001) (Table 5). The positive NRI and IDI values confirm that the inclusion of hematological markers leads to improved patient reclassification and discrimination, reinforcing the value of this multidimensional predictive approach.

**Table 5:** The improvement of basic clinical data combined with blood routine and coagulation function indicators in the diagnosis of aSAH with acute symptomatic hydrocephalus.

Models	NRI (continuous)		NRI (categorical)		IDI	
	Estimate (95% CI)	*P*	Estimate (95% *CI*)	*P*	Estimate (95% CI)	*P*
Basic	Ref.	–	Ref.	–	Ref.	–
Basic + WBC + M + TT	0.490 (0.268–0.712)	<0.001	0.170 (0.058–0.282)	0.003	0.070 (0.041–0.098)	<0.001

Basic: age, gender, drinking, smoking, hypertension, coronary atherosclerotic heart disease, diabetes mellitus, cerebral infarction.

WBC, white blood cell; M, monocytes; TT, thrombin time; NRI, net reclassification improvement; IDI, integrated discrimination improvement; Ref.: reference.

## Discussion

aSAH remains a devastating disease with high morbidity and mortality (18), and survivors are also at a high risk of long-term functional impairment (19). Hydrocephalus is a common cause of neurological deterioration after aSAH. In this study, our results demonstrated that acute symptomatic hydrocephalus in aSAH patients was positively associated with WBC and M in blood routine, and negatively associated with coagulation function parameters TT. Compared with basic clinical data (including age, sex, smoking history, drinking history, hypertension, diabetes, coronary heart disease, and cerebral infarction), the combination of “basic clinical data + WBC + M + TT” was more effective in predicting acute symptomatic hydrocephalus in aSAH patients. In addition, high levels of WBC and M may lead to a greater risk of aSAH with acute symptomatic hydrocephalus in males and patients aged <60. However, high levels of TT may lead to a lower risk of aSAH complicated with acute symptomatic hydrocephalus in women and patients ≥60 years of age, which was a protective factor.

WBC are composed of granulocytes, monocytes, and lymphocytes, playing crucial roles in both innate and adaptive immunity to defend against pathogen invasion (20). Studies have shown that WBC infiltration in the walls of intracranial aneurysms promoted aneurysm formation, growth, and rupture, processes considered to be inflammatory in nature (21). Further histopathological research found a positive correlation between the number of WBC in the aneurysm wall and the aneurysm's fragility, with a higher WBC count associated with an increased risk of rupture (22). Additionally, studies reported that clinical grade differences in aSAH patients were associated with peripheral blood WBC elevation; however, peripheral WBC count increases had not been directly linked to poor prognosis (23). It remains unclear whether WBC elevation was merely an accompanying phenomenon of disease severity or a biomarker of the underlying inflammatory process triggered by the initial intracranial injury. In our study, we found that high levels of WBC may lead to a greater risk of aSAH complicated with acute symptomatic hydrocephalus in males and patients <60 years of age as a risk factor, which may have been due to a combination of factors. Biologically, hormonal levels differ between males and females (24), potentially influencing immune system responses and making them more prone to WBC elevation in response to certain stimuli. Furthermore, men under 60 in modern society often faced significant psychological stress, and prolonged stress may lead to endocrine dysfunction, which in turn affected immune system function. Stress-induced release of norepinephrine could have promoted the proliferation of hematopoietic stem cells in the bone marrow, leading to increased WBC release into peripheral circulation (25). Therefore, we speculate that chronic psychological stress may have resulted in hormonal imbalances, affecting the generation and function of WBCs, thereby increasing the risk of acute symptomatic hydrocephalus in aSAH patients. While these hormonal and stress-related mechanisms are proposed to explain the sex- and age-specific associations, they remain speculative and require validation in dedicated mechanistic or prospective studies investigating hormone levels and stress biomarkers. Future studies could investigate the specific mechanisms linking WBC elevation to acute symptomatic hydrocephalus in aSAH patients, and the role of male and age-related factors in this process, providing more targeted strategies for clinical treatment and prevention.

M are a key component of the innate immune system, playing a crucial role in the occurrence, regulation, and resolution of inflammation through cytokine production and antigen presentation (26). M accumulated in the CSF of aSAH patients (27), and elevated peripheral blood M levels at admission had been associated with the development of hydrocephalus (28). These findings consistently supported the role of M in both primary and secondary brain injury in the early stages of aSAH, but the underlying pathophysiological mechanisms remained unclear. In fact, the innate immune system was rapidly activated during the early phase of SAH, and immune cells were transported from the periphery to the brain across the blood-brain barrier (29). In our study, we found that aSAH complicated with acute symptomatic hydrocephalus was positively associated with M in blood routine, and a high level of M may lead to a greater risk of aSAH complicated with acute symptomatic hydrocephalus in males and patients <60 years old. This phenomenon may be related to the interaction of multiple physiological and immune factors. Gender differences (sexual dimorphism) in the immune system existed, with males and females exhibiting differences in immune responses (30). Research by Chen showed that the function of M was sex-specific, with absolute M counts generally higher in males than in females (31). Additionally, androgens had been shown to modulate the activity and function of M (32). Therefore, we hypothesize that in male patients with aSAH, M may have been more easily activated and involved in the inflammatory response. For patients aged <60 years, their bodies were in a relatively active physiological state, with faster metabolism and potentially stronger immune responses. When aSAH occurred, this heightened immune response may have led to excessive activation and accumulation of M, thereby increasing the risk of hydrocephalus development. These explanations regarding sex-specific monocyte function and age-related immune reactivity are plausible hypotheses based on existing literature but are currently unsupported by direct experimental evidence (e.g., sex hormone measurements or functional immune assays) in our cohort and should be considered speculative.

TT is the standardized prothrombin plasma clotting time, primarily reflecting the time required for fibrinogen to convert to fibrin after the addition of standardized thrombin. It could also have served as a monitoring indicator for thrombolytic and anticoagulant therapies. A study by Wan demonstrated that the intraventricular injection of thrombin could simulate hydrocephalus following SAH (33). Therefore, thrombin generated during the coagulation process after SAH may have played a significant role in the formation of hydrocephalus. Recent studies also showed that FIB, TT, and activated partial thromboplastin time (APTT) were associated with the Fisher grade of aSAH (34), suggesting that coagulation function may have played an important role in the clinical presentation and complications of aSAH. Our study found that the development of acute symptomatic hydrocephalus in aSAH patients was negatively associated with TT, and TT may have been a protective factor, lowering the risk of hydrocephalus in female patients and those aged ≥60 years. Specifically, estrogen in females was thought to have a protective effect on vascular endothelial cells (35), potentially influencing the function of the coagulation system. Thus, we hypothesize that estrogen may have modulated endothelial cell function, affecting platelet aggregation and coagulation factor activity, which could have resulted in prolonged TT and a reduced risk of acute symptomatic hydrocephalus in aSAH patients. For patients aged ≥60 years, as aging progresses, various physiological functions declined, including reduced vascular elasticity and changes in coagulation function (36). Aging may have impaired the vascular wall's ability to repair damage, leading to a relatively slower coagulation process and prolonged TT. This physiological change may have inhibited the formation of hydrocephalus. However, the influence of gender and age differences on the development of acute symptomatic hydrocephalus in aSAH patients requires direct experimental confirmation (e.g., measuring estrogen levels, assessing vascular repair capacity, or characterizing age-related coagulation profiles), and the proposed mechanisms remain conjectural. Additionally, systematic analysis of coagulation function differences across age groups in aSAH patients remained lacking. Therefore, future research should further explore the potential pathophysiological mechanisms underlying these differences.

In this study, we found that the development of acute symptomatic hydrocephalus in aSAH patients was positively associated with WBC and M levels in the blood routine, and negatively associated with the coagulation function parameter TT. Furthermore, the combined prediction model of “basic clinical data + WBC + M + TT” showed better predictive performance for acute symptomatic hydrocephalus in aSAH patients. The scientific and logical basis for these findings could be discussed from several perspectives. First, WBC and M, as markers of inflammation, may have reflected a robust immune response during the acute phase of aSAH. Inflammation in aSAH patients could have lead to increased blood-brain barrier permeability, which in turn affected CSF circulation and absorption, thus increasing the risk of hydrocephalus (4, 37). Therefore, the positive correlation between elevated WBC and M levels and the development of acute symptomatic hydrocephalus in aSAH patients was biologically plausible. Second, TT, as an important coagulation function indicator, reflected the coagulation state of the blood. Coagulation dysfunction played a significant role in the pathophysiological process of aSAH (33). A shorter TT may have indicated a hypercoagulable state, which predisposed the patient to thrombosis, potentially impairing cerebral blood circulation and increasing the risk of hydrocephalus. Therefore, the negative correlation between TT and acute symptomatic hydrocephalus in aSAH patients was also consistent with the underlying pathophysiological mechanisms. Moreover, by combining basic clinical data (such as age, sex, and medical history) with WBC, M, and TT, a more comprehensive risk assessment could be made. This multidimensional prediction approach integrated the patient's general health status, inflammatory response, and coagulation function, providing more accurate high-risk warning information for clinical use. By analyzing these factors together, high-risk patients could be identified earlier, enabling the development of personalized intervention strategies (such as anti-inflammatory treatment, anticoagulant therapy, and CSF drainage). This could reduce complications and improve patient outcomes. In conclusion, this multi-layered predictive approach, by considering various biomarkers, offered a more comprehensive set of evaluation indicators for clinical practice. Future research could further explore the interrelationships between these markers and their specific roles in the pathogenesis of acute symptomatic hydrocephalus in aSAH patients. Deeper investigation into these mechanisms may further refine predictive indicators, enhance early diagnosis and intervention capabilities, and provide stronger support for improving the prognosis of aSAH patients.

**Figure 3:**
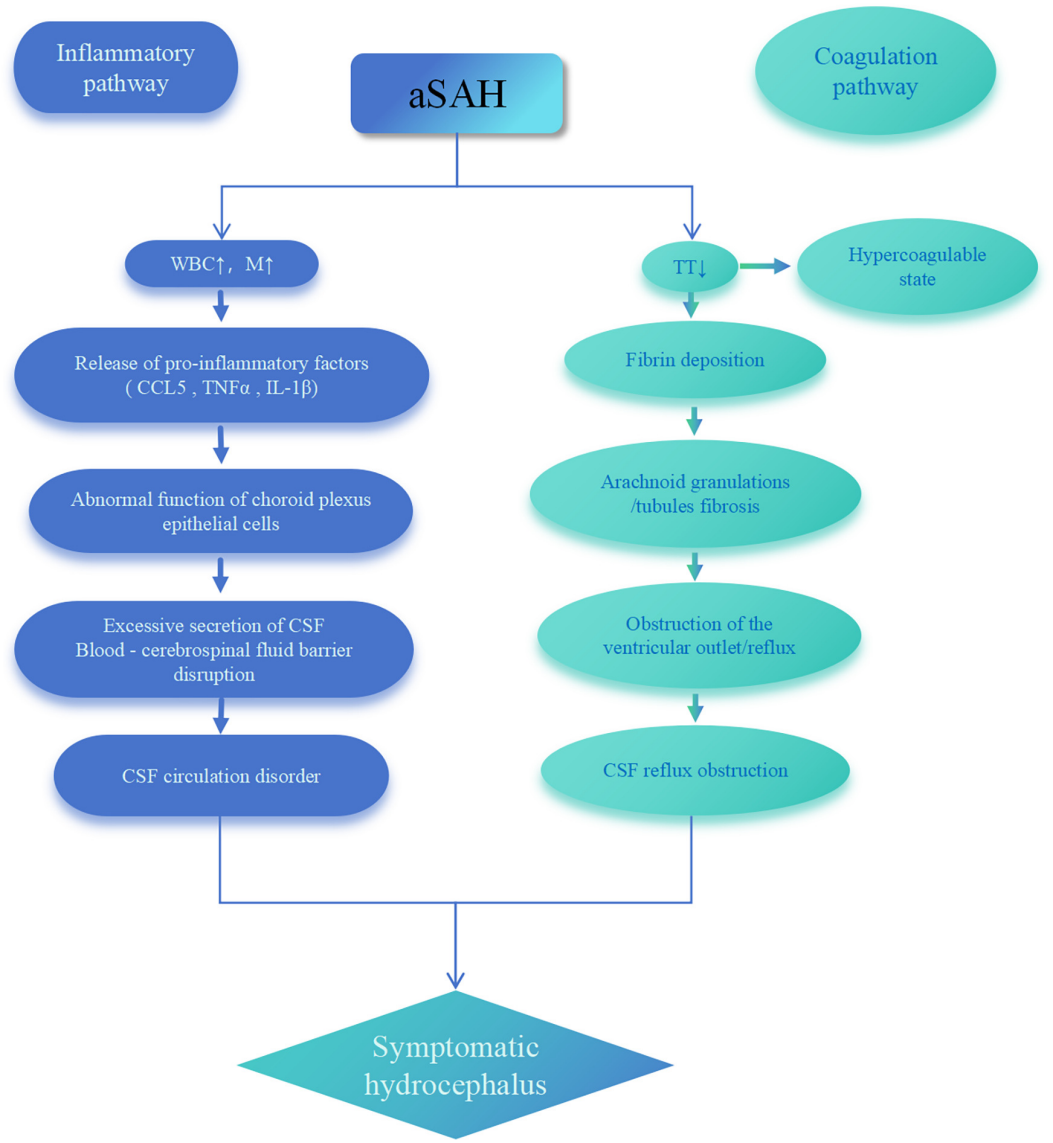
Pathophysiological model of acute symptomatic hydrocephalus following aSAH. aSAH, aneurysmal subarachnoid hemorrhage; WBC, white blood cell; M, monocytes; TT, thrombin time; CSF, cerebrospinal fluid; CCL5, C-C motif chemokine ligand 5; TNFα, tumor necrosis factor alpha; IL-1β, interleukin-1 beta.

Studies report that C-C motif chemokine ligand 5 (CCL5) mediates leukocyte infiltration into the ventricular system, triggering inflammatory responses that disrupt CSF circulation and induce hydrocephalus (38). M play a pivotal role in post-SAH choroid plexus inflammation: they differentiate into macrophages and release pro-inflammatory factors [Tumor Necrosis Factor alpha (TNFα), Interleukin-1 beta (IL-1β)], directly impairing choroid plexus epithelial cell function. This leads to excessive CSF secretion and blood-cerebrospinal fluid barrier damage, ultimately promoting hydrocephalus development (39). Concurrently, hyperactivation of exogenous coagulation in CSF synergizes with inflammatory responses to disrupt CSF circulation pathways, inducing chronic hydrocephalus (40). Wan et al. demonstrated that intraventricular thrombin injection replicates hydrocephalus following subarachnoid hemorrhage (33). Therefore, based on the above evidence, we propose the pathophysiological model in Figure 3: After aSAH, elevated WBC and M trigger the release of pro-inflammatory factors (CCL5, TNFα, IL-1β), causing choroid plexus epithelial cell dysfunction that drives CSF hypersecretion and blood-cerebrospinal fluid barrier disruption. Simultaneously, shortened TT reflects a hypercoagulable state that promotes fibrin deposition and fibrosis in arachnoid granulations, mechanically obstructing CSF reflux. These synergistic pathways ultimately induce acute symptomatic hydrocephalus. This model provides mechanistic explanation for the predictive value of WBC, M, and TT.

Several prior studies have examined the relationship between peripheral blood markers and hydrocephalus after aSAH. For example, Cuoco reported that admission monocyte counts predicted shunt-dependent hydrocephalus (28), and Chaudhry highlighted elevated CSF CCL5 levels as predictors of poor outcomes (38). Moreover, Mohme characterized monocyte activation profiles in aSAH patients (27). Unlike these single-biomarker or CSF-focused approaches, our study innovatively integrates WBC, M, and TT into a multidimensional predictive model. However, our research has limitations. First, as a single-center retrospective analysis, it risks selection bias and limited external validity, lacking the robustness of prospective multicenter trials. This may involve incomplete/inaccurate data. Second, we did not perform serial measurements of WBC, M, and TT, preventing analysis of temporal trends to refine prediction. Future work should track daily biomarker trajectories (e.g., days 1–7 post-aSAH) with time-series modeling. Third, while statistically informative, the cohort remains limited; larger prospective studies are needed. Fourth, although we propose inflammatory/coagulation pathways and sex/age effects based on literature, direct experimental validation (e.g., hormone assays, choroid plexus immunohistochemistry, immune cell studies) is absent. Cross-sectional admission data cannot establish causality or distinguish biomarkers as drivers vs. epiphenomena of early ventricular changes. Fifth, we acknowledge that unmeasured confounders (e.g., aneurysm location, hemorrhage volume) may influence hydrocephalus risk. However, this study focused on admission hematological markers due to their early accessibility and clinical utility for rapid risk stratification. Future models should integrate imaging parameters. To address these, multicenter prospective studies must validate our model across diverse populations, explore additional biomarkers, and combine serial imaging with mechanistic assays to clarify causal pathways.

## Conclusions

The WBC, M and TT within 24 h of admission in aSAH patients can be used to predict the occurrence of acute symptomatic hydrocephalus. Future research could further explore the interrelationships between these markers and their specific roles in the pathogenesis of acute symptomatic hydrocephalus in aSAH patients.

## Data Availability

The original contributions presented in the study are included in the article/Supplementary Material, further inquiries can be directed to the corresponding authors.
